# Editorial: Women in developmental and reproductive toxicology: 2021

**DOI:** 10.3389/ftox.2022.1026314

**Published:** 2022-10-03

**Authors:** Jennifer J. Adibi, Sofie Christiansen, Suzanne E. Fenton, Alison Holloway, Heather B. Patisaul

**Affiliations:** ^1^ Department of Epidemiology, Graduate School of Public Health, University of Pittsburgh, Pittsburgh, PA, United States; ^2^ National Food Institute, Technical University of Denmark, Kongens Lyngby, Denmark; ^3^ Division of the National Toxicology Program, National Institute of Environmental Health Sciences, Durham, NC, United States; ^4^ Reproductive Biology Division, Department of Obstetrics and Gynecology, McMaster University, Hamilton, ON, Canada; ^5^ Center for Human Health and the Environment, NC State University, Raleigh, NC, United States

**Keywords:** mentor, women’s health, gender, fetal, mammary gland, placenta

Women remain a minority group in toxicology, both as lead investigators and in terms of using the tools of toxicology to understand environmental influences on women’s health. While the fields of toxicology and environmental medicine are diversifying, underrepresented groups, including women and women of color, face unique challenges. When unmet, this can lead to isolation, lack of support, career dissatisfaction, and ultimately higher rates of attrition. This special issue was therefore conceived to highlight the work of women toxicologists to emphasize their unique mentorship and support needs, and to maximize career success. Given this focus, the scientific work primarily addresses women and child health (i.e., pregnancy and health of the next generation). This special issue contains two original data papers, a mini-review, and a perspective piece on what is needed to support and mentor women in what remains a male-dominated field.

In their mini-review, Björvang and Mamsen provide evidence that many man-made chemicals, including persistent organic pollutants (POPs) accumulate in the placenta in human fetuses. Notably, some pollutants accumulate to a higher degree in male fetal tissues and their associated placentas suggesting that gestational exposure can be sexually dimorphic. Their call for greater attention on sexual differences in adverse health outcomes in toxicological studies aligns with efforts by NIH and others to consider sexual dimorphism and include sex as a biological variable in all biomedical research.

Blake et al. also focused on the placenta in their paper and took a high-throughput toxicity screening approach to examine the impact of 42 unique per- and polyfluoroalkyl (PFAS) compounds on trophoblast cell viability and function. Using the human placental trophoblast JEG-3 cell line, the team found that some common PFAS impaired cell migration, induced oxidative stress, and impaired cellular response to xenobiotic stress. These data are critically significant given that several of these PFAS are ubiquitous environmental contaminants and that nearly every water and drinking water source contains complex cocktails of PFAS, making exposure unavoidable. Multiple outcomes including adverse pregnancy outcomes in women, higher risk of dyslipidemia, elevated cholesterol and low birth weight have already been associated with PFAS exposure. These data further illustrate the potential for PFAS to disrupt placental and fetal health. The findings in the paper suggest the placenta is a direct target of PFAS exposure with trophoblast cell gene expression and function as sensitive markers of exposure, while the mechanism(s) of action of PFAS toward placental trophoblasts remain unknown.

Sunscreen is widely used to prevent skin damage including skin cancer. Despite their protective effects on skin, several chemical sunscreens have been shown to be endocrine disrupting once absorbed. Absorbance is higher than originally thought. Here, a team led by Matouskova et al. built on their prior work showing that perinatal exposure to the common sunscreen ingredient, oxybenzone, disrupts mammary gland development in mice. Mammary gland effects in rodents have previously shown to occur at low doses of several chemicals, demonstrating the exquisite sensitivity of the female. Using exposure levels that are relevant to humans, the team found evidence of stromal abnormalities including the increased presence of mast cells in mammary glands of adult female mice exposed perinatally. Their work adds to a rapidly growing literature showing that oxybenzone has endocrine disruptive effects even in low doses, in humans, in mice and other sensitive species.

Finally, Swanson discusses strategies for recruiting, mentoring, coaching, and supporting women at all levels in the field of toxicology. Importantly, she emphasizes the need to teach young trainees how to mentor as formal training is rarely available and this is a critical component to the survival of women in science. She addresses the need for long-term and holistic relationships that address the ways in which the challenges of women differ from those of their male counterparts. This includes psychological barriers that come with isolation, imposter syndrome, and the experience of managing simultaneously complex needs of the household and the family, along with the needs of the research group. She essentially argues for a fundamental change in culture around mentoring and evaluation of women in science that would be a step forward from the traditional model that centers the values and priorities of a culture established decades ago, when patriarchy and gender roles were unquestioned and unchallenged. For example, structured mentoring programs that take on different configurations including group mentoring or mentoring networks might be more effective in helping women navigate hierarchies and political barriers in the institution. Swanson discusses the distinction between mentoring, coaching (support to learn a specific skill in a short time frame and with feedback) and sponsorship (championing a woman to develop competencies in knowing why, knowing how, and knowing whom), which are all essential to success. This triad is also referred to as mentors being allies, ambassadors and master-teachers. Dr. Swanson’s commentary is timely as, with the pandemic, rates of burnout, decreased productivity, and loss of quality mentors are at epidemic levels. Reconceiving what effective mentorship looks like will help diversify the field, recruit, and retain talent.

Many of the tenets established in this manuscript also translate to mid-career investigators who may no longer be a recipient of “investment” and are ready to take on the challenges in positions of leadership and upper management. For this group of women, Swanson introduces the idea of reverse mentoring to update skills and help drive cultural change. Although diversity, equity, inclusion, and accessibility efforts are being enhanced and supported by funding institutions, there is still an abundance of senior-level positions held by males in academia and industry.

Enhanced efforts to hire and mentor a diverse workforce, with women/women of color in positions of leadership may inspire future generations of women to remain in science.

In conclusion, the four contributions in this special issue of “Women in Developmental and Reproductive Toxicology: 2021” provide examples of excellent toxicology and emphasize sexually dimorphic effects, the role of the placenta, mammary gland effects and the overall developmental and reproductive toxic effects of POPs and other man-made chemicals on humans and animal species. The perspective piece gives food for thoughts on what is needed now and, in the future to support and mentor women in the field of Toxicology.

